# Stay on Target: Reengaging Cancer Vaccines in Combination Immunotherapy

**DOI:** 10.3390/vaccines9050509

**Published:** 2021-05-15

**Authors:** Benjamin Wolfson, S. Elizabeth Franks, James W. Hodge

**Affiliations:** Laboratory of Tumor Immunology and Biology, Center for Cancer Research, National Cancer Institute, Bethesda, MD 20892, USA; ben.wolfson@nih.gov (B.W.); frankssae@nih.gov (S.E.F.)

**Keywords:** immunotherapy, combination therapy, cancer, vaccine, clinical trial, adjuvant cytokines, multifunctional

## Abstract

Effective treatment of established tumors requires rational multicombination immunotherapy strategies designed to target all functions of the patient immune system and tumor immune microenvironment. While these combinations build on the foundation of successful immune checkpoint blockade antibodies, it is increasingly apparent that successful immunotherapy will also require a cancer vaccine backbone to engage the immune system, thereby ensuring that additional immuno-oncology agents will engage a tumor-specific immune response. This review summarizes ongoing clinical trials built upon the backbone of cancer vaccines and focusing on those clinical trials that utilize multicombination (3+) immuno-oncology agents. We examine combining cancer vaccines with multiple checkpoint blockade antibodies, novel multifunctional molecules, adoptive cell therapy and immune system agonists. These combinations and those yet to enter the clinic represent the future of cancer immunotherapy. With a cancer vaccine backbone, we are confident that current and coming generations of rationally designed multicombination immunotherapy can result in effective therapy of established tumors.

## 1. Introduction

Throughout the 20th century, chemotherapy rapidly evolved from using single agents as a monotherapy to highly integrated multicombination therapy, utilizing multiple chemotherapeutic agents combined with surgery, radiation, molecularly targeted therapies and immuno-oncology agents [1]. Each agent is chosen to target different mechanisms driving tumor growth, survival and recurrence while minimizing toxicity in the patient. In the 21st-century, cancer treatment has undergone a paradigm shift moving away from prioritizing toxic chemotherapeutic agents and radiation towards immuno-oncology agents and immunotherapy. In cancer patients, tumors escape immune surveillance both by mutations eliminating their own antigens and through the production of tumor cell-derived factors that disrupt the cancer-immunity cycle, leading to decreased effector cell generation, migration and activity [2]. The goal of immunotherapy is to activate the immune system, reversing tumor progression along the “three Es” of cancer immunoediting [3], pushing it from immune escape to elimination.

To date, the most mature immuno-oncology agents are immune checkpoint blockade (ICB) antibodies, which enable an already present immune infiltrate to promote tumor killing. While seven ICB agents have been FDA approved in multiple cancer indications as monotherapy [4], they do not function in all tumor types or patients. This is in part due to the absence of effective immune infiltration in these tumors. These non-inflamed tumors (“cold” tumors) present clinicians with problems that are incapable of being addressed through current ICB tactics [5,6]. For effective cancer control, immunotherapy strategies must follow the same path chemotherapies did a century before ‒ moving from monotherapy towards rationally designed combination therapy with multiple immuno-oncology agents, each selected to target different functions of the patient immune system, tumor and tumor microenvironment (TME), resulting in an ideal antitumor microenvironment.

First-generation cancer vaccine-based combination immunotherapy involved using single cancer vaccines combined with cytokines to activate the immune system. While not rationally designed, these treatments were often combined with standard-of-care therapies that vary widely on a patient-by-patient and indication-by-indication basis, many of which have an immunogenic impact in addition to cytotoxic effect [7]. The development of effective ICB agents brought the second generation of vaccine-based combination immunotherapy combining multiple immuno-oncology agents. Numerous second-generation combination immunotherapy trials are underway, combining checkpoint blockade antibodies with each other and with cancer vaccines. Clinical trials are currently entering the third generation of combination immunotherapy, utilizing strategies that encompass the 4 Es of effective combination immunotherapy: combining agents that *engage* the immune system through the induction of tumor-antigen specific immunity, *expand* the specific tumor immune response, *enable* the immune response to ensure prolonged and persistent antitumor activity, and ensure an *evolving* immune response, preventing tumor escape [7,8].

As the success of immunotherapy strategies depends on the physical presence of immune infiltrate, and specifically on the existence and presence of tumor antigen-specific cytotoxic T cells, cancer vaccines are an integral component of combination immunotherapy strategies moving forward. Through generating tumor-specific effector cells, cancer vaccines *engage* the antitumor immune response and provide a critical foundation on which other immuno-oncology agents can build. Despite this, recent phase III clinical trials of the sialyl-TN keyhole limpet hemocyanin vaccine in metastatic breast cancer [9], MVA-5T4 vaccine in metastatic renal cancer [10] and PROSTVAC vaccine in metastatic castration-resistant prostate cancer (mCRPC) [11] have shown that cancer vaccine monotherapy strategies lack clinical efficacy. While disappointing, these trials demonstrated an overall lack of toxicity for monotherapy vaccine and the ability of cancer vaccines to induce tumor-specific cytotoxic T cells. Some possible reasons for the lack of success of cancer vaccines as a monotherapy include selecting target antigens, adjuvant components, trial design and biomarker availability. Multiple vaccine vectors have proven safe; therefore, it is possible that using more immunogenic tumor antigens, combinations of tumor antigens, or neoantigens would be more effective. Furthermore, some vaccines have displayed clinical benefit with increased time on trial and more vaccinations [10]. It is also likely that certain patient populations are more receptive to therapeutic vaccination, suggesting increased use of biomarkers and patient selection may demonstrate a clinical benefit in select groups [12].

Moreover, these monotherapy vaccine failures clearly indicate the necessity of multicombination strategies. Cancer vaccines play an important role in immunotherapy by *engaging* a patient’s immune system; however, additional agents must also be employed to capitalize on the presence of these tumor antigen-specific immune cells. Moving forward, these combinations will utilize new multifunctional molecules, immune agonists, adoptive cell therapy, as well as novel vaccine technologies, such as personalized and neoantigen vaccines.

This review focuses on next-generation and combination strategies that build upon the backbone of cancer vaccines. We have previously reviewed ongoing trials utilizing well-characterized agents in vaccine combination immunotherapy [7]; however, these agents will likely be insufficient for effective tumor control. Herein we examine the current state of next-generation immuno-oncology agents and survey the ongoing multi-agent clinical trials that utilize them to truly address all aspects of tumor immunity.

## 2. Second-Generation Combination Therapy

While first-generation combination therapy strategies have demonstrated some efficacy, the FDA approval of the cytotoxic T-lymphocyte‒associated protein 4 (CTLA-4) blocking antibody ipilimumab in 2011 [13] has ushered in an era of rationally designed combination immunotherapy strategies. There are currently seven FDA-approved ICB therapies, all targeting either the molecules CTLA-4, programmed death protein 1 (PD-1) or programmed death-ligand 1 (PD-L1) [4]. CTLA-4 is expressed on T cells and functions by competitively binding B7 ligand on antigen-presenting cells, preventing the CD28-B7 interaction necessary for T-cell activation. By blocking this interaction, CTLA-4 blocking antibodies prevent CTLA-4‒mediated inhibition of T-cell activation [14,15]. Similarly, PD-1 is expressed on T cells, B cells and NK cells, and after binding, PD-L1 reduces proliferation, cytotoxicity and cytokine secretion. PD-L1 is expressed on some tumors but also on various immune cells [16,17].

### 2.1. Combining Multiple Immune Checkpoint Blockade Antibodies

Many patients treated with CTLA-4 and PD-1/PD-L1 blocking antibodies demonstrate prolonged response rates with low toxicity; however, ICB efficacy is restricted to certain cancer indications, and not all patients respond [18]. One strategy to combat this is combining multiple immune checkpoint blockade antibodies targeting both CTLA-4 and the PD-1/PD-L1 axis simultaneously. While CTLA-4 inhibition primarily engages the immune system through promoting T-cell activation in the lymph nodes and preventing regulatory T cell (Treg)‒mediated dendritic cell (DC) suppression, blockade of the PD-1/PD-L1 signaling axis abrogates inhibition of natural killer (NK) and effector T-cell activation in peripheral tissues [4]. Therefore, it was theorized that targeting multiple mechanisms of engaging the immune system would expand the efficacy of ICB. There are approximately 20 completed or ongoing clinical trials investigating ICB combination in melanoma, colorectal cancer, renal cell carcinoma, small cell and non-small cell lung carcinoma (NSCLC) and others.

Further, the combination of nivolumab (anti-PD-1) and ipilimumab (anti-CTLA-4) is FDA-approved in DNA mismatch repair-deficient/microsatellite instability-high metastatic colorectal cancer metastatic melanoma and renal cell carcinoma. In colorectal cancer, the overall response rate of this combination was 58%, with progression-free survival (PFS) at 60% and overall survival (OS) at 74% after 24 months [19]. In melanoma, the median OS was over 60 months in patients receiving nivolumab and ipilimumab, compared with 36.9 months in patients receiving nivolumab alone and 19.9 months in patients with ipilimumab alone [20]. In renal cell carcinoma, clinicians observed a 75% 18 month OS rate and 42% objective response rate (ORR) in nivolumab plus ipilimumab, compared with 60% OS and 27% ORR in patients receiving sunitinib, the current standard of care [21]. It should be noted that similar to immune checkpoint blockade monotherapy, this combination has not proven successful in all cancer indications. A recent phase III clinical trial of pembrolizumab plus ipilimumab in metastatic NSCLC (NCT03302234) found that patients receiving the combination had an OS of 21.4 months while those receiving pembrolizumab alone had an OS of 21.9 months. Furthermore, patients receiving pembrolizumab plus ipilimumab reported greater toxicity, leading to death in 13.1% of patients versus 7.5% of patients in the monotherapy arm [22]. These findings clarify that, even in combination, the efficacy of immune checkpoint blockade therapy is not universal.

### 2.2. Combination Checkpoint Therapy with Vaccine

While combination checkpoint therapy is effective in these cancer models, it still requires the presence of a tumor-specific immune infiltrate, explaining why patients who harbor “cold” tumors are resistant to monotherapy or combination immune checkpoint blockade. Numerous trials are ongoing investigating the efficacy of combining one checkpoint molecule with a tumor antigen vaccine. We have identified 11 that have expanded to multicombination therapy through using dual checkpoint blockade and vaccine. Four employ conventional cancer vaccines (Table 1); seven are investigating next-generation neoantigen vaccine technology (Table 2).

### 2.3. Conventional Cancer Vaccines

Cancer vaccines should target highly immunogenic proteins that have expression restricted to cancer cells and are necessary for tumor survival [23]. Conventional vaccine targets are either tumor-associated antigens, which are self-antigens upregulated in tumor cells, or tumor-specific antigens, such as oncogenes or oncoviral antigens, which are restricted to the tumor tissue.

We have identified one currently recruiting phase Ib study using the OTSGC-A24 peptide vaccine combined with nivolumab and ipilimumab in gastric cancer (NCT03784040). OSTGC-A24 is a peptide vaccine cocktail targeting the tumor-specific antigens FOXM1, DEPDC1, KIF20A, URLC10, and the angiogenesis promoter VEGF [24]. While there are currently no results from this trial, a previous phase I/Ib study of OSTGC-A24 in patients with advanced gastric cancer found that it was safe and observed significantly increased cytotoxic T lymphocyte (CTL) responses with a median PFS of 1.7 months and OS of 5.7 months [24]. Two additional trials are investigating combining nivolumab and ipilimumab with peptide vaccines. In colorectal and pancreatic cancer, there is one study utilizing a pooled mutant-KRAS peptide vaccine (NCT04117087), and in fibrolamellar hepatocellular carcinoma (FLC), a DNAJB1-PRKACA fusion kinase peptide vaccine (NCT04248569). KRAS is the most frequently mutated oncogene in cancer and is a primary driver of pancreatic, colorectal and lung cancers [25]. This makes mutated KRAS an ideal potential vaccine target. Similarly, the DNAJB1-PRKACA fusion kinase has been recently identified as the driver of FLC, a recently identified rare disease that primarily affects patients in their 20 s [26]. The dependence of FLC on DNAJB1-PRKACA combined with high expression of PD-1 and PD-L1 in the tumor and TME of FLC [27] makes combining ICB and a tumor vaccine an ideal therapeutic strategy.

Finally, we have identified a phase I/II study combining an mRNA vaccine, BI 1361849, with durvalumab and tremelimumab in NSCLC (NCT03164772). Durvalumab is an anti-PD-1 antibody that is FDA-approved in bladder and lung cancer, while tremelimumab is an anti-CTLA-4 antibody that has yet to be approved for use. BI 1361849 encodes for six NSCLC tumor-associated antigens, MUC1, survivin, NY-ESO-1, 5T4, MAGE-C2 and MAGE-C1. While a phase Ib trial of BI 1361849 with radiation treatment in stage IV NSCLC recently showed that BI 1361849 was well tolerated and found increased antigen-specific antibody levels to at least one BI 1361849 antigen in 80% of patients, multiple BI 1361849 antigens in 52% of patients and increased functional T cells in 40% of patients [28], no data have been published detailing the multiple ICB combined with BI 1361849 [29].

### 2.4. Neoantigen Vaccines

Immune checkpoint blockade has proven to be highly effective in patients whose tumors exhibit significant mutational burdens. Tumor mutations frequently lead to the increased presence of tumor neoantigens, and ICB activation of cytotoxic T cells specific to these neoantigens may be the reason for increased patient response [30]. Furthermore, neoantigens are more immunogenic than tumor-associated antigens, and therefore, may be ideal vaccine targets [31,32]. However, there are still substantial barriers in the translation of neoantigen vaccine research to effective clinical therapy. To create personalized neoantigen vaccines, the neoepitopes must be identified, determined to be highly expressed in the tumor, and found to be immunogenic, meaning they are processed and presented correctly on human leukocyte antigen (HLA) molecules and recognized by T cells. After the design of a neoantigen vaccine, it must be rapidly manufactured before it can be delivered to a patient. Neoantigen vaccines can be produced using various platforms, including long synthetic peptides, RNA, DNA, and mature dendritic cells that have been exposed to neoantigen peptides or RNA. While each has advantages and disadvantages, vaccines on all platforms are being investigated in the clinic [33].

There are currently ~100 clinical trials investigating neoantigen vaccines as monotherapy and an additional ~20 clinical trials combining neoantigen vaccines with a single agent [34]. We have identified seven clinical trials combining neoantigen vaccines with multiple immuno-oncology agents. These trials encompass a diverse set of neoantigen vaccines; however, none have reported results. Where possible, we will discuss the foundational data of these trials.

We have identified one clinical trial utilizing the personal neoantigen vaccine NeoVax combined with nivolumab and ipilimumab in patients with melanoma (NCT03929029). NeoVax is a long peptide vaccine that targets up to 20 neoantigens and is admixed with Poly-ICLC, a proinflammatory agonist for TLR3 and MDA5 [35]. In a phase Ib trial in glioblastoma, patients were given five priming vaccines followed by two booster vaccines. Only 3/8 patients completed the booster regimen; the other five experienced disease progression and discontinued therapy. The median PFS was 7.6 months, and OS was 16.8 months [36]. Furthermore, a long-term follow-up of a clinical trial examining melanoma patients was recently published showing durable immunity, and after 55 months, 8/8 patients remained alive. Melanoma recurrence occurred in 5/8 patients. Two experienced a complete response after pembrolizumab treatment. One was treated with surgical resection, and two developed metastatic diseases. The researchers observed that in all eight patients, 28–59 months after vaccination, 68% of the specific CD4 T-cell responses and 59% of the epitope-specific CD8 T-cell responses recorded at week 16 remained detectable, indicating a persistent immune response [37].

Another synthetic long peptide vaccine is being investigated in a phase II clinical trial combined with durvalumab, tremelimumab and Nab-paclitaxel to evaluate the differential clinical response of the addition of the neoantigen vaccine (NCT03606967) [38]. While no data have been released concerning the efficacy of this vaccine in the clinic, using patient-derived xenografts, the researchers demonstrated the efficacy of their neoantigen prediction method. They found that neoantigen-specific CD8 T cells were capable of inhibiting tumor growth in vivo [39]. Moreover, paclitaxel has been demonstrated to induce immunogenic modulation, which may further engage the immune system and promote an antitumor response [40].

Finally, we identified a clinical trial combining a long peptide vaccine with the anti-PD-1 molecule toripalimab and granulocyte macrophage-colony stimulating factor (GM-CSF) in melanoma; however, no additional information is available concerning this trial or vaccine (NCT04072900).

Two trials are ongoing, utilizing a prime/boost strategy combined with nivolumab and ipilimumab. Prime/boost is an important vaccination strategy wherein patients are “primed” with vaccines using one vector model, followed by multiple subsequent boosts utilizing a different vector. It is primarily used when the “prime” vaccine is a vector for which a repeated immune response is not possible, such as the vaccinia virus [41]. In the identified trials, the prime vaccines, GRT-C901 or GRT-C903, are a modified chimpanzee adenovirus, followed by boost vaccines with self-amplifying mRNA in lipid nanoparticles (GRT-C902, GRT-3904). In a phase 1/2 study (NCT03639714), both vaccines contain personalized neoantigens, whereas, in a related trial (NCT03953235), the vaccines contain shared neoantigens. Twelve patients have been treated so far: in NCT03639714, six patients with gastroesophageal adenocarcinoma, NSCLC or colorectal cancer were treated with personalized neoantigen vaccines, and in NCT03953235, six patients with NSCLC, colorectal cancer or pancreatic cancer were treated with shared neoantigen vaccines. In the personalized neoantigen trial, of four analyzed patients, all showed neoantigen-specific CD8 T-cell response to multiple neoantigens after the primer vaccine, and 2/3 patients analyzed had further increases after boost vaccination. In the shared neoantigen trial, 1/3 evaluable patients had a robust, specific CD8 T-cell response. These findings demonstrate the potential immunogenicity of these vaccine formulations and the prime/boost model [42].

One other identified clinical trial (NCT03532217) involves multiple vaccines. However, rather than combining multiple neoantigen vaccines, it involves combining a neoantigen DNA vaccine with ipilimumab, nivolumab and the vaccine PROSTVAC V/F. PROSTVAC V/F is a diversified prime/boost vaccine containing transgenes for prostate-specific antigen and TRICOM, the three T-cell costimulatory molecules B7-1, ICAM-1 and LFA-3. It consists of a vaccinia priming vaccine followed by a fowlpox boost [43]. Currently, 18 patients with metastatic colorectal cancer have been recruited. The primary objective is safety and tolerability, with secondary objectives of failure-free survival, milestone survival at 2 years, PSA response and radiographic PFS. However, no data have as yet been released [44].

A second neoantigen DNA vaccine clinical trial is currently ongoing in hepatocellular carcinoma. This phase I/II trial combines pembrolizumab with the personalized neoantigen DNA vaccine GNOS-PV02 and INO-9012, a plasmid encoding the cytokine interleukin (IL)-12, which promotes T helper cell differentiation and IFN***γ*** production and enhances T cell and NK cell cytotoxicity [45] (NCT04251117). While no data are currently available concerning this combination or the efficacy of GNOS-PV02, INO-9012 is in an additional phase I/II clinical trial in glioblastoma combined with the DNA vaccine INO-5401, which expresses the cancer antigens human telomerase reverse transcriptase (hTERT), Wilms’ tumor gene 1 (WT-1) and prostate-specific membrane antigen (PSMA). Interim results have shown an acceptable safety profile of this combination, supporting using INO-9012 combined with GNOS-PV02 in hepatocellular carcinoma [46].

## 3. Third-Generation Immunotherapy Combinations

Third-generation immunotherapy strategies are defined by the rational combination of multiple agents, each targeting a different immune system *function to engage, expand, enable and evolve the antitumor immune response*. To do so requires novel agents and strategies outside of conventional checkpoint blockade antibodies and vaccines as well as new, multifunctional fusion molecules capable of performing several functions in addition to targeting the tumor more effectively. Herein, we will discuss current clinical trials exemplifying this new paradigm, either through combination with multifunctional fusion molecules or through broad, rationally designed combinatorial trials delivering multiple agents.

### 3.1. Adjuvant Cytokines

Adjuvant cytokines are one of the most commonly used classes of immuno-oncology agents. Cytokines are pleiotropic in nature with many overlapping, redundant and independent mechanisms of action and cellular targets. They can be broadly broken down by their function of either stimulating or suppressing cells of the immune system and other cell types. Tumor cells can exploit immunostimulatory cytokines and secrete immunosuppressive cytokines to evade immune detection/clearance [47]. Systemic administration of cytokines in various cancer settings has had some success, but overall toxicity limits the breadth of their efficacy. The most commonly used adjuvant cytokines are GM-CSF and IL-2. However, numerous additional cytokines, including IFN⍺, IFN***γ***, IL-12, IL-15, IL-18 and IL-21, have demonstrated efficacy as vaccine adjuvants [48].

GM-CSF supports antigen processing and presentation [49], and IL-2 stimulates T-cell growth and differentiation after stimulation [50]. While IL-2 and IFN⍺ have demonstrated efficacy as monotherapy agents and gained FDA approval across several indications, they fell out of favor due to low response rate and high toxicity [51]. Cytokine treatment remains an effective combination partner for vaccines, and novel agents that increase cytokine half-life and tumor localization through using targeted fusion molecules or gene therapy vectors are being actively studied. Two of these, ALT-803 (also called N-803) and NHS-IL-12, are actively in clinical trials.

ALT-803 is an IL-15/IL-15Rα superagonist, with a substantially prolonged half-life compared to recombinant IL-15, enhancing both T and NK cell activity [52]. Our laboratory has recently shown that ALT-803 can rescue NK cell cytotoxicity that had been inhibited through exposure to transforming growth factor-β (TGF-β) [53]. We have identified eight trials utilizing ALT-803 combined with vaccine and multifunctional agents (Table 3); however, over a dozen clinical trials are utilizing ALT-803 in various combinations across multiple indications (QUantum Integrative Lifelong *Trial* (QUILT) trials; clinicaltrials.gov (accessed on 1 May 2021)).

Finally, NHS-IL-12 is a fusion molecule designed to reduce the toxicity associated with systemic IL-12 administration. IL-12 is a potent proinflammatory cytokine produced by professional antigen-presenting cells that exerts paracrine effects on CD8 T cells, NK cells and NKT cells, effectively regulating both innate and adaptive immunity. IL-12 can also act in an autocrine fashion, driving increased antigen processing machinery of DCs [54]. Although administration of recombinant IL-12 (rIL-12) displayed promising clinical activity in phase I trials [55], it is accompanied by an unacceptable level of adverse events (reviewed here; [56]). NHS-IL-12 is a fusion of two IL-12 homodimers fused to the NHS76 antibody. NHS76 is a human IgG1 monoclonal antibody that binds exposed histones of DNA, allowing targeting of this antibody to necrotic regions of the TME [57,58]. This targeted delivery of IL-12 to the tumor reduces toxicities observed with systemic treatment with rIL-12 and can increase immune cell infiltrate.

### 3.2. Multifunctional Molecules

Conventional combination immunotherapy regimens have been hampered by the need to separately infuse multiple agents, inconveniencing patients and increasing the risk of toxicity. To rectify this, multifunctional molecules have been developed that are the fusion of two or more different functional structures in one molecule. In addition to reducing the time patients are in treatment chairs, multifunctional molecules may also possess the ability to exhibit their mechanism of action in a more targeted, tumor-directed manner.

Although there are several multifunctional immuno-oncology agents in preclinical and clinical development [59], there is one multifunctional agent currently in clinical trials combined with cancer vaccines: bintrafusp alfa (formerly M7824). Bintrafusp alfa is a bifunctional molecule targeting both an immune checkpoint receptor and an additional pathway contributing to tumor immune escape. Bintrafusp alfa consists of a monoclonal antibody targeting PD-L1 with the receptor for TGF-β fused to its C-terminus [60]. TGF-β can exert suppressive functions on immune cells in the TME, resulting in decreased responsiveness of T cells (as indicated by reduced perforin, granzyme A and granzyme B expression) and NK cells (as evidenced by decreased activating receptor expression [61,62] and granzyme expression [63,64]). The bifunctionality of this molecule targets areas with high PD-L1 expression, such as the TME, sequestering TGF-β at that site and effectively reducing the tumors’ immunosuppressive response in an autocrine or paracrine manner.

Eight clinical trials are ongoing, combining one or more multifunctional molecules with cancer vaccines. Of these, two combine solely a multifunctional molecule and vaccine (NCT03315871 and NCT04432597, they will not be discussed in detail for brevity), while the remaining six combine a multifunctional molecule with vaccine and additional immunomodulating agents (Table 3).

A phase Ib trial in patients with advanced-stage or metastatic breast cancer aims to identify a new combination of immunotherapy drugs to enhance clinical benefit (BN-Brachyury, Entinostat, Adotrastuzumab Emtansine and M7824 in Advanced Stage Breast Cancer (BrEAsT); NCT04296942). The treatment backbone of this trial includes the cancer vaccine targeting the tumor-associated antigen Brachyury (MVA-BN-Brachyury/FPV-Brachyury) combined with bintrafusp alfa (M7824; experimental arm 1). Patients enrolled in experimental arm 2 will receive MVA-BN-Brachyury/FPV-Brachyury, bintrafusp alfa and ado-trastuzumab emtansine (TDM-1), and patients in experimental arm 3 will receive the combination mentioned above plus the histone deacetylase (HDAC) inhibitor entinostat. Entinostat, a selective inhibitor of class I and IV HDAC enzymes resulting in a block of cell proliferation and differentiation, and ultimately leading to apoptosis [65], has shown promising results in phase I and II trials (reviewed here; [66]). The primary outcomes of this trial include ORR (in triple-negative breast cancer (TNBC) and HER2+) and safety, with secondary measures, including PFS (TNBC and HER2+) and immune correlative analysis. No results have been posted as of this writing.

We have identified an additional phase I/II trial treating patients with metastatic TNBC or p16-negative head and neck squamous cell carcinoma (HNSCC) with bintrafusp alfa, the cancer vaccine MVA-BN-CV301/FPV-CV301, and SX-682, a small molecule inhibitor of CXCR1/2 (NCT04574583). MVA-BN-CV301/FPV-CV301 is a poxviral-based vaccine encoding for tumor-associated antigens MUC1 and CEA, as well as TRICOM [67]. This vaccine is a prime/boost model similar to PROSTVAC-V/F as described above. SX-682 inhibits two key chemokine receptors responsible for the migration of myeloid-derived suppressor cells (MDSCs). Inhibition of CXCR1/2 in animal models has shown a reduction in MDSC infiltration into the TME, increased T cell accumulation, and enhancement of T cell-targeted immunotherapies [68]. Additionally, SX-682 and M7824 act synergistically in murine models of breast and lung cancer by reducing MDSC and enhancing infiltration of T cells into the TME [69]. Further rationale for using this small molecule inhibitor comes from the clinic where low CXCR1/2 expression is correlated with increased survival compared to patients with high CXCR1/2 expression. This trial aims to evaluate the safety, tolerability, and maximum tolerated dose (MTD) of SX-682 combined with M7824 and MVA-BN-CV301/FPV-CV301 vaccines in patients with advanced or metastatic solid tumors. Primary outcome measures include identifying a recommended phase II dose (RP2D) and ORR, followed by secondary outcomes measures of disease control rate (DCR) and PFS.

Patients with advanced or metastatic human papillomavirus (HPV)‒associated malignancies represent an unmet clinical need for active treatment of these tumors that are poorly palliated by standard therapies. We have identified a phase I/II trial investigating the combinatorial effects of an HPV vaccine (PDS0101), bintrafusp alfa and NHS-IL-12 that is actively recruiting (NCT04287868). While patients in a phase I trial of bintrafusp alfa showed a significantly higher response rate (34.9%) than patients treated with single-agent PD-1 inhibitors alone (15–20%) (NCT02517398), there is room for improvement. PDS0101 employs the Versamune^®^ platform, possessing a proprietary compilation of HPV16 antigens, and was well tolerated in a phase I, open-label, dose-ranging study (NCT02065973). The primary outcome is to evaluate ORR, with secondary outcomes to determine the safety of the combination therapy of PDS0101, NHS-IL-12 and bintrafusp alfa, in addition to PFS, OS and adverse events (AEs). No results have been posted as of this writing.

The remaining two trials combining multifunctional molecules with cancer vaccines combine at least two multifunctional molecules, resulting in simultaneously targeting several immune system functions. The first phase is a phase II trial in patients with metastatic or refractory/recurrent small bowel and colorectal cancers (NCT04491955). While these patients infrequently respond to checkpoint inhibitors (reviewed here; [70]), preclinical evidence suggests that combining multiple immune systems‒targeting agents may improve antitumor efficacy and clinical benefit. This trial aims to evaluate the ORR in subjects with advanced checkpoint naïve microsatellite stable (MSS) small bowel or colorectal cancers treated with CV301, a cancer vaccine targeting CEA and MUC1, combined with ALT-803 (N-803), bintrafusp alfa and NHS-IL-12. Accrual for this trial began in September 2020; no results have been posted as of this writing.

Finally, we have identified a clinical trial employing M7824, ALT-803, MVA-BN-Brachyury and FPV-Brachyury, and the indoleamine 2,3-dioxygenase-1 (IDO1) inhibitor epacadostat in metastatic castration-resistant prostate cancer (mCRPC) [71]. Low success rates with checkpoint blockade therapy in mCRPC are hypothesized to be the result of poor immune cell infiltration to the tumor. Therefore, combining checkpoint blockade with a cancer vaccine and additional immunomodulating therapies could lead to more robust and durable clinical responses in this setting. IDO1 has multiple immune suppressive effects that include, but are not limited to, induction of cell cycle arrest, apoptosis, reduction in NK cell activation [72], and activation of regulatory T cells [73].

### 3.3. Adoptive Cell Therapy

Another immunotherapeutic strategy that has proven successful for a wide variety of malignancies is adoptive cell therapy (reviewed extensively here; [74,75,76]), with over 100 clinical trials investigating various cell transfer protocols. The greatest success with adoptive cell therapy occurs in liquid cancer, with much work continuing in solid malignancies. We will discuss trials that combine adoptive cell therapy with vaccines and at least one additional agent.

In multiple myeloma patients receiving autologous stem cell transplantation (ASCT), treatment results in an extended event-free survival (EFS) in 20%–40% of cases [77,78], indicating the potential for improvement. In a phase I/II clinical trial for patients with multiple myeloma, investigators attempted to improve the results of ASCT while producing an effective antitumor immune response to eliminate residual disease (NCT00834665). Fifty-four patients were enrolled in the trial’s two arms, Arm A and Arm B. Both arms received ASCT, transfer of autologous ex vivo stimulated T cells, immunization with a pneumococcal conjugate vaccine (PCV) and GM-CSF. Patients positive for the HLA-A2 antigen were enrolled in Arm A and also received a tumor-antigen peptide vaccine containing hTERT and survivin, called hTERT/survivin. Interestingly, patients receiving hTERT/survivin had inferior EFS compared to patients enrolled in Arm B, who received PCV alone (25% and 65%, respectively). The authors attribute this inferiority to the fact that only 36% of patients in Arm A mounted a response to the vaccine as well as to differences in patient and treatment-related factors, such as percentage of plasma cells in the bone marrow at the time of enrollment, thalidomide maintenance and others [79]. When the data are corrected for these differences, the 3 years projected survival for all 54 patients is 83%, with no significant difference between treatment arms. Interestingly, although patients in Arm A mounted greater immune responses to the hTERT/survivin compared to patients receiving idiotype vaccines, the magnitude is still significantly lower than for patients receiving a microbial vaccine.

This same group recently completed a phase II trial investigating combination immunotherapy and ASCT in myeloma (NCT01245673), specifically attempting to increase tumor-specific immunity. Because only 1/3 of patients responded to the hTERT/survivin vaccine in their previous trial, the investigators added Poly-ICLC (a TLR3 agonist) to a MAGE-A3‒derived multi-peptide vaccine. Importantly, 76% of patients had an observable immune response as evidenced by T-cell cytokine analysis in response to vaccination. The 2 year EFS and OS for all patients were 56% and 74%, respectively. However, 62% of patients had observed clinical responses, while 35% had partial responses or stable disease [80]. This study showed it is possible to generate a robust vaccine-specific autologous T-cell response, providing a further rationale to investigate whether these T cells result in clinical benefit.

Adoptive cell therapy with NK cells has also proven effective, and several immortalized NK cell therapeutic products are in combination trials with vaccines (Table 3). Activated NK (aNK, NK-92) [81] is an immortalized NK cell line that has proven safe in clinical trials in diverse solid tumors/sarcomas, acute myeloid leukemia and hematological malignancies following relapse after autologous hematopoietic stem cell transplantation (HSCT) [82]. NK-92 has been further engineered into the cell line termed “high-affinity NK cell” (haNK), which expresses the high-affinity CD16/FcγRIIIA (158 V) allele, which induces antibody-dependent cellular cytotoxicity (ADCC) mediated through the Fc region of IgG1 antibodies, resulting in cell lysis [83]. haNK cells are in phase I trials (NCT03027128, NCT03027128); however, no results have been posted. Further information regarding NK cell transfer in treating cancers can be found in a previously published review [82].

### 3.4. Immune Cell Agonists

Unlike checkpoint blockade molecules that stimulate the immune system by blocking negative signaling interactions, agonistic antibodies exert their function by inducing signaling of the activating receptor for which they are specific. The tumor necrosis factor receptor superfamily (TNFRSF) contains many receptors responsible for the development, survival and function of immune cells, including OX40, CD40, FasR, 4-1BB, among others [84]. Studies interrogating the efficacy of monoclonal antibodies specific for costimulatory receptors, such as CD40 (dacetuzumab, CP-870,893 and lucatumumab [85,86,87,88]), OX40 (9B12 [89], BMS-986,178 [90]) and LAG3 (IMP321 [91]), are currently underway. Targeting these receptors with agonistic monoclonal antibodies could be beneficial in generating more robust antitumor immune responses.

OX40 is found on the surface of T cells and is modulated upon T-cell activation, resulting in increased proliferation, effector function and survival of antigen-specific T cells [89]. These factors suggest that OX40 is an attractive target for treating cancer. Administration of an OX40 agonist as monotherapy in preclinical murine models resulted in tumor volume control and enhanced antitumor T-cell activity when combined with checkpoint blockade therapy [92,93,94], providing a rationale for such a combination to be used in the clinic.

We have identified one trial combining an agonistic antibody with a cancer vaccine. This phase I/IIa trial is investigating the experimental medication BMS-986178 combined with nivolumab and/or ipilimumab in patients with advanced solid cancers (NCT02737475). BMS-986178 is a fully-humanized IgG1 agonistic antibody specific for OX40 that has shown early evidence of monotherapy activity in preclinical murine models that is increased when combined with checkpoint blockade therapy [95]. Preliminary data from this trial demonstrate that BMS-986178 as a monotherapy or combined with nivolumab and/or ipilimumab is well tolerated with no dose-limiting toxicities (DLTs) or discontinuation due to study treatment [79]. Although earlier data showed that BMS-986178 monotherapy resulted in increased proinflammatory cytokines and higher proliferation and activity of CD4 and CD8 T cells when combined with either anti-PD-1 or anti-CTLA-4 [96], this was not recapitulated in this trial upon interrogation of intratumoral or peripheral CD8 T cells [90]. Results obtained here suggest that the responses observed were not greater than what would have been projected for nivolumab or ipilimumab treatment alone. Investigators attribute the lack of clinical signal to a heterogeneous patient population, timing and sequence of dosing, as well as optimum receptor occupancy required for response. Strategies to increase response rates to agonists combined with checkpoint blockade therapy include cancer vaccines, radiation, Toll-like receptor agonists, etc. As this trial continues, Arm 9 aims to enroll patients with TNBC and identify the clinical response of BMS-986178, nivolumab, cyclophosphamide and DPV-001, an autophagosome cancer vaccine containing 25 putative cancer antigens, damage-associated molecular patterns (DAMPs), heat shock proteins, and TLR2, 3, 4, 7 and 9 agonists [97]. This arm is open for enrollment, and no results have been posted at the time of this writing.

## 4. Conclusions

**Figure 1:**
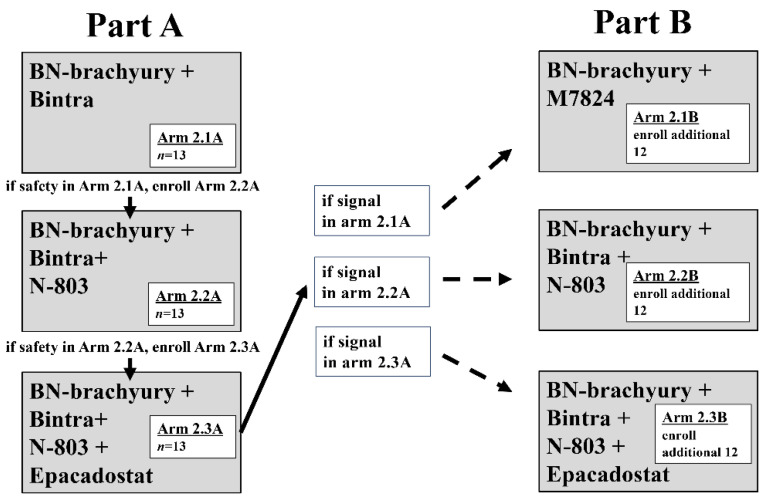
Quick efficacy seeking trial design trial schema. During part A, enrollment to arms 1.1 and 2.1A begins simultaneously. Arm 1.1 is a dose-finding arm for N-803 combined with Bintrafusp alfa (Bintra), open to all solid tumors. After arm 2.1A completes accrual and safety of the combination has been demonstrated, and N-803 dosing has been determined from arm 1.1, arm 2.2A begins accrual. After arm 2.2A completes accrual and safety of the combination has been demonstrated, enrollment to arm 2.3A begins. Each of the 3 arms enrolls a total of 13 patients during part A. At the completion of part A, if there is a positive safety signal and a positive efficacy signal in arm 2.1A, 2.2A, or 2.3A, part B will begin. To further assess efficacy, arms in which an activity signal was observed (arms 2.1B, 2.2B, and/or 2.3B) may expand to a total of 25 patients. During part B, patients are randomized among all open arms to avoid selection bias.

As clinical research enters the third generation of combination immunotherapy, it is clear that the progression of immunotherapy in the 21st century will match that of chemotherapy in the 20th century. The number of clinical trials employing rationally designed multicombination therapy involving conventional therapeutic agents, proven immuno-oncology agents, novel immuno-oncology agents and multifunctional molecules on a vaccine backbone is growing rapidly. Results are expected soon. However, as combination immunotherapy is applied more widely and utilized combinations expand, it is important to keep in mind the potential treatment-induced toxicities [98]. Furthermore, conventional trial design results in significant periods required to determine the safety and efficacy of individual agents before combining them. A recent trial discussed above utilizes a unique modified experimental design to expedite the investigation of multiple immunotherapy regimens (combinations of BN-Brachyury, bintrafusp alfa, ALT-803 and epacadostat) and rapid clinical signal assessment, termed Quick Efficacy Seeking Trial or QuEST. QuEST1 comprises two arms, with sequential accrual into each arm and each sequential arm adding a new immuno-oncology agent. In part A, arms 1.1 (ALT-803 + bintrafusp alfa) and 2.1 (bintrafusp alfa + BN-Brachyury) assess MTD followed by the addition of more agents if safety is demonstrated. If safety, tolerability, and clinical signal are observed in part A, the cohorts will be expanded in part B, allowing for the rapid increase in the number of patients treated (Figure 1) [71]. This clinical strategy, and others like it, will prove instrumental in pushing patient treatment into the next generation of rationally designed multicombination immunotherapy. These strategies will ensure that all agents and combinations will be fully interrogated and that early winners will be able to expand quickly, reaching and helping more patients. With therapeutic cancer vaccines as their foundation, we are confident that current and coming generations of multicombination immunotherapy will result in effective therapy of established tumors.

## Figures and Tables

**Table 1:** Combining multiple checkpoint blockade antibodies with conventional cancer vaccines.

Clinical Trial #	Trial Name	Indication	Status	Trial Phase	*n*	Treatments
NCT03784040	Nivolumab, Ipilimumab and OTSGC-A24 Therapeutic Peptide Vaccine in Gastric Cancer—a Combination Immunotherapy Phase Ib Study	Gastric cancer	Recruiting	I	40	OTSGC-A24 Nivolumab Ipilimumab
NCT04117087	Pooled Mutant KRAS-Targeted Long Peptide Vaccine Combined With Nivolumab and Ipilimumab for Patients With Resected MMR-p Colorectal and Pancreatic Cancer	Colorectal cancer Pancreatic cancer	Recruiting	I	30	KRAS peptide vaccine Nivolumab Ipilimumab
NCT04248569	DNAJB1-PRKACA Fusion Kinase Peptide Vaccine Combined With Nivolumab and Ipilimumab for Patients With Fibrolamellar Hepatocellular Carcinoma	Fibrolamellar hepatocellular carcinoma	Recruiting	I	12	DNAJB1-PRKACA peptide vaccine Nivolumab Ipilimumab
NCT03164772	Phase 1/2 Study of Combination Immunotherapy and mRNA Vaccine in Subjects With NSCLC	Metastatic non-small-cell lung cancer	Recruiting	I/II	56	BI 1361849 Durvalumab Tremelimumab

**#**: NCT: clinicaltrials.gov identification number. OTSGC-A24: peptide cancer vaccine-targeting tumor antigens FOXM1, DEPDC1, KIF20A, URLC10, VEGFR1. KRAS: Kirsten rat sarcoma virus oncogene. DNAB1-PRCACA: fusion kinase that is major driver of fibrolamellar hepatocellular carcinoma. BI 1361849: mRNA vaccine targeting tumor-associated antigens MUC1, survivin, NY-ESO-1, MAGE-C2, MAGE-C1.

**Table 2:** Combining multiple checkpoint blockade antibodies with neoantigen vaccines.

Clinical Trial #	Trial Name	Indication	Status	Trial Phase	*n*	Treatments
NCT03929029	Neoantigen-based Personalized Vaccine Combined With Immune Checkpoint Blockade Therapy in Patients With Newly Diagnosed, Unmethylated Glioblastoma	Glioblastoma	Active, not recruiting	I	3	NeoVax Nivolumab Ipilimumab
NCT03606967	Testing the Addition of an Individualized Vaccine to Nab-Paclitaxel, Durvalumab and Tremelimumab and Chemotherapy in Patients With Metastatic Triple Negative Breast Cancer	Breast cancer	Recruiting	II	70	Personalized synthetic long peptide vaccine Durvalumab Tremelimumab Nab-paclitaxel Carboplatin
NCT04072900	A Personalized NeoAntigen Cancer Vaccine Combined With Anti-PD-1 in Melanoma	Melanoma	Recruiting	I	30	Peptide neoantigen vaccine Anti-PD-1 GM-CSF
NCT03953235	A Study of a Personalized Cancer Vaccine Targeting Shared Neoantigens	Non-small cell lung cancer Colorectal cancer Pancreatic cancer	Recruiting	I/II	144	GRT-C903 GRT-C904 Nivolumab Ipilimumab
NCT03639714	A Study of a Personalized Neoantigen Cancer Vaccine	Non-small cell lung cancer Colorectal cancer Gastroesophageal adenocarcinoma Urothelial cancer	Recruiting	I/II	214	GRT-C901 GRT-C902 Nivolumab Ipilimumab
NCT03532217	Neoantigen DNA Vaccine in Combination With Nivolumab/Ipilimumab and PROSTVAC in Metastatic Hormone-Sensitive Prostate Cancer	Metastatic hormone-sensitive prostate cancer	Recruiting	I	20	PROSTVAC-V/F Neoantigen DNA vaccine Nivolumab Ipilimumab
NCT04251117	GNOS-PV02 Personalized Neoantigen Vaccine, INO-9012 and Pembrolizumab in Subjects With Advanced HCC	Hepatocellular carcinoma	Recruiting	I/II	24	GNOS-PV02 INO-9012 Pembrolizumab

**#**: NCT: clinicaltrials.gov identification number. NeoVax: personalized peptide vaccine. PROSTVAC-V/F: recombinant vaccinia and fowlpox vaccines expressing prostate-specific antigen, LFA-3, ICAM-1, B7.1. GRT-C901: personalized modified chimpanzee adenovirus neoantigen vaccine. GRT-C902: boost vaccine matching GRT-C901 containing self-amplifying mRNA in lipid nanoparticles. GRT-C903: modified chimpanzee adenovirus neoantigen vaccine targeting shared neoantigens. GRT-C904: boost vaccine matching GRT-C903 containing self-amplifying mRNA in lipid nanoparticles. GM-CSF: granulocyte-macrophage colony-stimulating factor. GNOS-PV02: personalized neoantigen DNA vaccine. INO-9012: DNA plasmid encoding IL-12.

**Table 3:** Third-generation combination immunotherapy.

Clinical Trial #	Trial Name	Indication	Status	Trial Phase	*n*	Treatments
NCT03315871	Combination Immunotherapy in Biochemically Recurrent Prostate Cancer	Prostate cancer	Recruiting	II	34	PROSTVAC-V/F Bintrafusp alfa
NCT04432597	HPV Vaccine PRGN-2009 Alone or in Combination With Anti-PDL1/TGF-Beta Trap (M7824) in Subjects With HPV Associated Cancers	HPV positive cancers	Recruiting	I/II	76	PRGN-2009 Bintrafusp alfa
NCT04296942	BN-Brachyury, Entinostat, Ado-trastuzumab Emtansine and M7824 in Advanced Stage Breast Cancer (BrEAsT)	Breast cancer	Recruiting	I	65	Brachyury-TRICOM Bintrafusp alfa Entinostat Ado-trastuzumab
NCT04574583	Phase I/II Trial Investigating the Safety, Tolerability, Pharmacokinetics, Immune and Clinical Activity of SX-682 in Combination With Bintrafusp Alfa (M7824 or TGF-beta “Trap”/PD-L1) With CV301 TRICOM in Advanced Solid Tumors (STAT)	Metastatic cancer Solid tumors	Recruiting	I/II	105	MVA-BN-CV301 FPV-CV301 Bintrafusp alfa SX-682
NCT04247282	Anti-PD-L1/TGF-beta Trap (M7824) Alone and in Combination With TriAd Vaccine and N-803 for Resectable Head and Neck Squamous Cell Carcinoma Not Associated With Human Papillomavirus Infection	Head and neck cancer	Recruiting	I/II	40	TriAdeno vaccine Bintrafusp alfa N-803/ALT-803
NCT04287868	Combination Immunotherapy in Subjects With Advanced HPV Associated Malignancies	HPV-positive cancers	Recruiting	I/II	40	PDS0101 Bintrafusp alfa NHS-IL12
NCT04491955	Phase II Trial of Combination Immunotherapy in Subjects With Advanced Small Bowel and Colorectal Cancers	Small bowel cancer Colorectal cancer	Recruiting	II	80	CV301 Bintrafusp alfa N-803 NHS-IL12
NCT03493945	Phase I/II Study of Immunotherapy Combination BN-Brachyury Vaccine, M7824, ALT-803 and Epacadostat (QuEST1)	Prostate cancer Advanced solid tumor	Recruiting	I/II	113	MVA-BN-Brachyury FPV-Brachyury Bintrafusp alfa Epacadostat ALT-803
NCT00834665	Phase I/II Clinical Trial Combining hTERT Tumor Vaccine & Autologous T Cells in Patients With Advanced Myeloma	Multiple myeloma	Completed	I	59	hTERT vaccine GM-CSF Pneumococcal conjugate vaccine T-cell infusion
NCT02737475	An Investigational Immuno-Therapy Study of Experimental Medication BMS-986178 by Itself or in Combination With Nivolumab and/or Ipilimumab in Participants With Solid Cancers That Are Advanced or Have Spread	Advanced solid cancer	Recruiting	I/II	207	DPV-001 BMS-986178 Nivolumab Ipilimumab Cyclophosphamide
NCT01245673	Combination Immunotherapy and Autologous Stem Cell Transplantation for Myeloma	Myeloma	Completed	II	28	MAGE-A3/GM-CSF Pneumococcal conjugate vaccine Lenalidomide Activated/costimulated autologous T cells
NCT03136506 NCT03329248 NCT03387085 NCT03387098 NCT03387111	QUILT-3.039 QUILT-3.060 QUILT-3.067 QUILT-3.070 QUILT-3.090	Pancreatic cancer Triple-negative breast cancer Squamous cell carcinoma	Active, not recruiting	I/II	3–173	ALT-803/N-803 Avelumab aNK haNK Nab-paclitaxel Bevacizumab Necitumumab Leucovorin Capecitabine 5-Fluorouracil Cyclophosphamide Oxaliplatin Cisplatin Lovaza SBRT Aldoxorubicin ETBX-051 ETBX-061 ETBX-011 GI-4000 GI-6207 GI-6301

**#**: NCT: clinicaltrials.gov identification number. PROSTVAC-V/F: recombinant vaccinia and fowlpox vaccines expressing prostate-specific antigen, LFA-3, ICAM-1, B7.1. Bintrafusp alfa: bifunctional molecule combining anti-PD-L1 antibody with TGF-βRII (TGF-β Trap). MVA-BN-Brachyury: modified vaccinia Ankara vaccine expressing tumor-associated antigen Brachyury. FPV-Brachyury: fowlpox vaccine expressing tumor-associated antigen Brachyury. ALT-803 (also called N-803): fusion molecule containing two molecules of IL-15 superagonist, two molecules of IL15α receptor and dimeric human IgG1 Fc. TriAdeno vaccine: adenoviral vaccine containing tumor-associated antigens CEA, MIC1 and Brachyury. PRGN-2009: gorilla adenoviral vaccine targeting HPV 16 and 18 oncoproteins E6 and E7. CV01 (FPV-CV301): poxvirus-based prime/boost vaccine targeting tumor-associated antigens CEA and MUC1. NHS-IL12: fusion immunocytokine containing two IL12 heterodimers and tumor necrosis targeting NHS76 antibody. SX-682: small molecule inhibitor of CXCR1/2 chemokine receptors. PDS0101: vaccine targeting HPV16 antigens. DPV-001: dendritic cell-targeted microvesicle vaccine. BMS-986178: OX-40 agonist monoclonal antibody. GM-CSF: granulocyte-macrophage colony-stimulating factor. MAGE-A3/GM-CSF: peptide vaccine targeting tumor antigen MAGE-A3 combined with immune adjuvant GM-CSF. aNK: activated natural killer (NK) cells. haNK: high-affinity NK cell. SBRT: stereotactic body radiation therapy. ETBX-051: adenoviral Brachyury vaccine. ETBX-061: adenoviral MUC1 vaccine. ETBX-011: adenoviral CEA vaccine. GI-4000: yeast-derived vaccine expressing mutant KRAS proteins. GI-6207: yeast-derived vaccine expressing CEA. GI-6301: yeast-derived vaccine expressing Brachyury.

## Data Availability

Not applicable.
